# Development and validation of a predictive scoring system for in-hospital mortality in COVID-19 Egyptian patients: a retrospective study

**DOI:** 10.1038/s41598-022-26471-w

**Published:** 2022-12-26

**Authors:** Mohamed AbdelSalam Elgohary, Asmaa Ali, Thanaa A. El-Masry, Hani Faidah, Farkad Bantun, Ahmad M. Elkholy, Jaklin S. Fahim, Nabila N. Elgamal, Mohamed Emam Mohamed, Mohamed G. Seadawy, Amro M. Helal, Michel De Waard, Hesham M. Shishtawy, Maisra M. El-Bouseary

**Affiliations:** 1 Department of Tropical Medicine, Military Medical Academy, Cairo, Egypt; 2 Department of Pulmonary Medicine, Abbassia Chest Hospital, MOH, Cairo, Egypt; 3 Department of Laboratory Medicine, School of Medicine, Jiangsu University, Zhenjiang, 212013 P. R. China; 4 Department of Pharmacology and Toxicology, Faculty of Pharmacy, Tanta University, Tanta, Egypt; 5 Department of Microbiology, Faculty of Medicine, Umm Al-Qura University, Makkah, Saudi Arabia; 6 Department of Tropical Medicine, Almaza Military Fever Hospital, Cairo, Egypt; 7 Department of Microbiology, Almaza Military Fever Hospital, Cairo, Egypt; 8 Biological Prevention Department, Ministry of Defense, Cairo, Egypt; 9 Department of Public Health, Almaza Military Fever Hospital, Cairo, Egypt; 10 Smartox Biotechnology, 6 rue des Platanes, 38120 Saint-Egrève, France; 11 L’institut du Thorax, INSERM, CNRS, Univ Nantes, F-44007 Nantes, France; 12 Université de Nice Sophia-Antipolis, LabEx “Ion Channels, Science & Therapeutics”, F-06560 Valbonne, France; 13 Egyptian Military Medical Services Administration, Cairo, Egypt; 14 Department of Pharmaceutical Microbiology, Faculty of Pharmacy, Tanta University, Tanta, Egypt

**Keywords:** Microbiology, Biomarkers, Health care, Medical research, Risk factors

## Abstract

SARS-CoV-2 virus has rapidly spread worldwide since December 2019, causing COVID-19 disease. In-hospital mortality is a common indicator for evaluating treatment outcomes. Therefore, the developing and validating a simple score system from observational data could assist in modulating the management procedures. A retrospective cohort study included all data records of patients with positive PCR for SARS-CoV-2. The factors that associated with mortality were analyzed, then allocation of potential predictors of mortality was executed using different logistic regression modeling, subsequently scoring system was developed from the most weighted predictors. The mortality rate of patients with COVID-19 pneumonia was 28.5% and 28.74%, respectively. The most significant factors that affected in-hospital mortality were old age (> 60 years), delay in hospital admission (> 4 days), high neutrophil/lymphocyte ratio “NLR” (> 3); higher computed tomography severity score; and CT-SS (> 20), in addition to using remdesivir and tocilizumab in the treatment protocol (P < 0.001 for all). The validity of the newly performed score was significant; the AUC was 85%, P < 0.001, and its prognostic utility was good; the AUC was 75%, P < 0.001. The prognostic utility of newly developed score system (EGY.Score) was excellent and could be used to adjust the treatment strategy of highly at-risk patients with COVID-19 pneumonia.

## Introduction

COVID-19 is a current pandemic disease caused by SARS-CoV-2 which is associated with serious health complications worldwide^1^. As of October 28, 2022, there were 626,337,158 confirmed cases of COVID-19, including 6,566,610 deaths, reported to the WHO^2^. The severity of COVID-19 infection ranges from asymptomatic manifestations to flu-like symptoms such as fever, headache, dry cough, dyspnea, mayalgia, and joint pain, eventually leading to fatal acute respiratory distress syndrome (ARDS)^3–5^. According to previous studies, the mortality rate for the most serious SARS-CoV-2 infections that require admission to the intensive care unit (ICU) ranges from 8.1 to 30% for hospitalized patients with SARS-CoV-2 pneumonia and up to 16% to 78% for patients who require admission to an ICU with critical care^4,6–8^.

According to previous studies, the COVID-19 outbreak is not uniform across nations, with notable variations in the proportion of serious diseases and case fatality rates^9^. Multicenter reports emphasize that patient-specific characteristics are important predictors of the presentation and consequences of COVID-19, even though the quality of healthcare services may be a factor in such variations^10^. Since the WHO officially declared a global pandemic in March 2020, there have been significant efforts to identify prognosticators that clinicians use to evaluate the risk at the early stage of the illness. This has helped to better tailor management strategies, assist decision-making, and promote health for COVID-19 patients by raising the therapeutic response, increasing the diagnostic accuracy, and lowering the case fatality rate^11^.

Various studies found that advanced age^12–15^, male gender^13^, and co-morbidities^13–15^ such as diabetes mellitus (DM), obesity, systemic hypertension (HTN), renal diseases, coronary artery disease^15^, and malignancy were risk factors for COVID-19 mortality. Beyond manifestations such as fever, cough^16^, haemoptysis^12^, dyspnoea^12,16^, unconsciousness^12^, clinical parameters such as elevated neutrophil/lymphocyte ratio^12,17^, and high levels of creatinine^18^, elevated lactate dehydrogenase (LDH)^12,14,15,18^, direct bilirubin^12^, and alanine amino-transferase (AST)^18^, which indicate early evidences to the severity of disease, an increased plasma level of biomarkers like d-dimer^15,18–20^, C-Reactive Protein (CRP)^21,22^, serum ferritin^18^, Interleukin-6 (IL-6)^18^, and procalcitonin (PCT) strengthens these findings^18–20^. The efficacy of particular antiviral and targeted immuno-modulatory therapies is still uncertain, therefore risk categorization and mortality prediction offer a logical strategy for allocating medical resources^23^.

The value of developing a score system for predicting the upcoming prognosis as mortality is vastly important for health care providers, especially doctors, to be more reliable and objective in evaluating their patients rather than being subjective^24,25^. Additionally, the recent publications in the medical field and beyond focus on the quality of the scoring system model and the methods of formation either through using strict and dedicated factors during statistical analysis steps or using machine learning programs^25–28^. Therefore, in order to estimate the probability of mortality in patients with COVID-19 pneumonia, forming a simple custom score could enhance the objective decision-making around treatment selection, as well as teach from trial and error in dealing with such a new disease. Our aim of the study was to develop and validate a simple scoring system to predict in-hospital mortality among patients with COVID-19 pneumonia within the first days of hospitalization, which was based mainly on different presenting symptoms, comorbidities, vital signs, and some laboratory data in addition to some local and affordable treatment options entered into the regression model. The multiple logistic regression model approaches were applied to our set of data to find the best equation that predicts the death probability. Furthermore, to measure the validity of that equation, a receiver operating characteristic curve was applied. Hence, the area under the curve expresses the goodness of that model in the prediction of mortality.

## Material and methods

### Study design and patient selection

**Figure 1:**
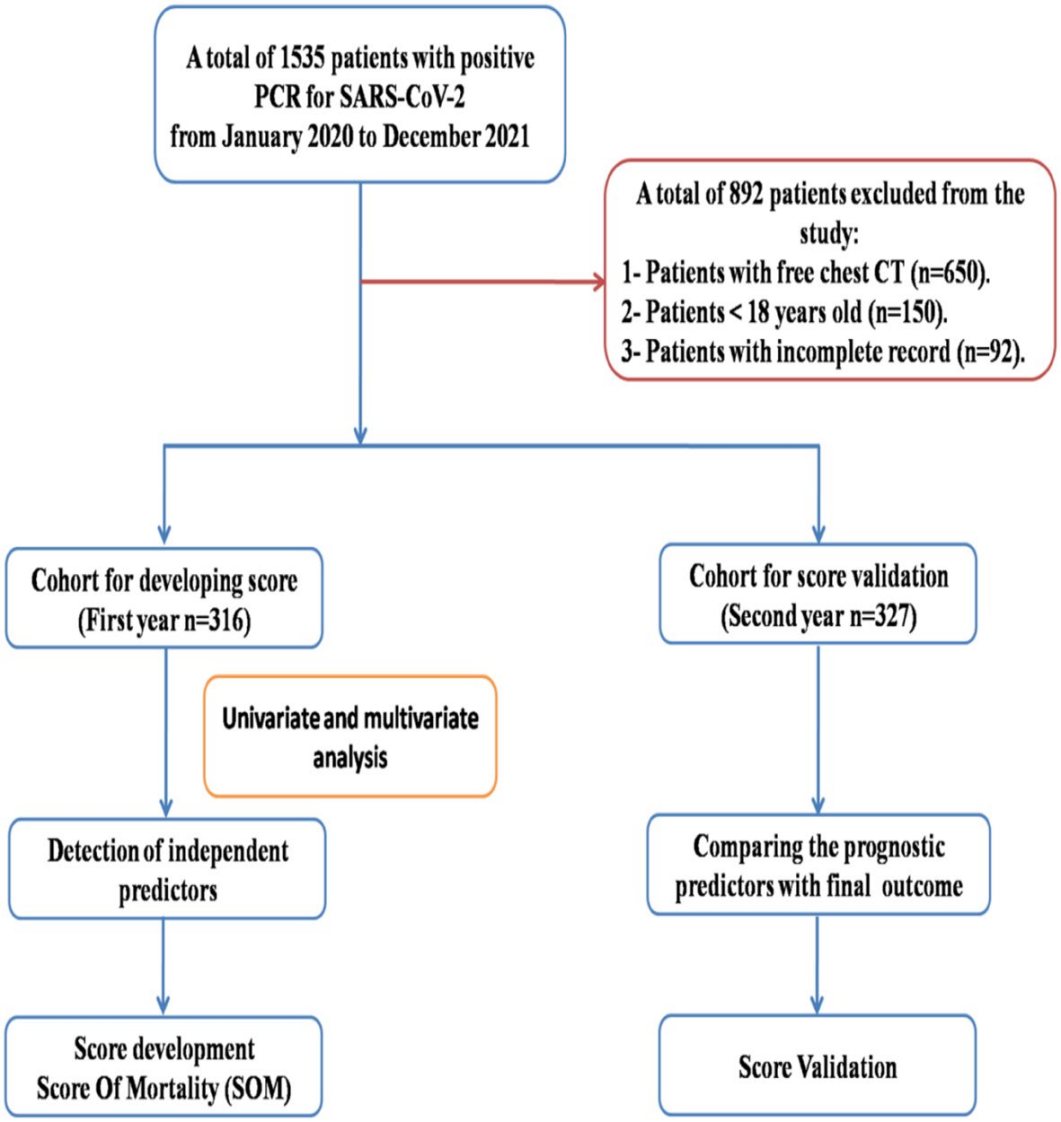
Flow chart for the study design.

A retrospective cohort study included all data records of patients with positive PCR for SARS-CoV-2 who was admitted to Almaza Military Hospital from January 2020 to the end of December 2021. A flow diagram showed the criteria of selected patients (Fig. 1). Hence, only 1535 patients had been considered as suggested candidates for primary study criteria. All of them had positive oropharangeal PCR tests in addition to CT chest. Those below 18 years old and those with free CT findings were excluded from the study. As well, cases with incomplete data records were also excluded. After informed written consent from all participants for publication of their data, the study has been approved by the Research Ethics Committee of the Faculty of Pharmacy, Tanta University (REC-TP code: TP/RE/012-21P-005) and the ethical committee office of the Medical Military Academy in agreement with the Helsinki Declaration Roles.

### Data collection

The data was extracted from the patient's files and included basic socio-demographic data in addition to the presenting symptoms. Only laboratory data that was conducted in the first 24 h after admission was considered. The evaluation of CT chest and quantifying CT-SS as regarding Yang et al.^29^, which was performed by two independent radiologists for reliability. The data about treatment protocol as regarding local guidelines was also included.

### Outcome

The outcome of desire was in-hospital mortality from COVID-19 pneumonia, which is defined as death during a period of admission to a hospital as a consequence of COVID-19 disease. Expectation of a short-term death after discharge from the hospital is also considered in-hospital mortality^30^.

### Statistical analysis

#### Building the predictive model and development of scoring system

All the data was collected and coded on an Excel sheet. The normality of data was examined using the Shapiro–Wilk test using SigmaPlot for Windows version 12.5. 0.38 (Systat Software, Inc., UK, 2011). The descriptive statistics were performed using Minitab 17.1.0.0 for Windows (Minitab Inc., 2013, Pennsylvania, USA). In the first step, all data was subjected to univariate analysis. Hence, the comparison between two means was done using an independent t-test, while the frequency comparison was made using the chi-square test. Regarding the Neyman-Pearson theory of classical statistics^31,32^, the critical value of a significant number was selected by the observer to guarantee that the type II errors were minimized as much as possible and circumvent the interrelationship between type I and type II errors. Additionally, Park*, 2013*^33^ mentioned that all variables could be implicated to test their correlation with the final outcome, provided that the log equation of fitness was acceptable; however, some authors preferred to select the variable of desire to be enrolled in the logistic equation after one step of simple hypothesis testing (univariate analysis)^34,35^, consuming that the margin of type II error was not so far away to accept the null hypothesis^36^. Therefore, in the current models, all factors with a p-value ≤ 0.35 were subjected to the second step, multivariate analysis. During building the logistic regression models, and to avoid multicollinearity, factors with VIF > 5 were eliminated. The goodness of fit for the regression model was performed using the Hosmer and Lemeshow test. Forward selection and backward elimination techniques were applied to select the best predictors for mortality. The Wald statistics numbers for each factor of interest were multiplied by the coefficient and divided by a constant value, and the result was rounded to the nearest integer number, taking into account the sign of the regression coefficient. According to the literature, all numerical data are transformed into categorical groups based on the distribution histogram and cumulative frequency or normal reference. The total score is then calculated from the submission of each individual score.

#### Score validation

The data for all factors implicated in the scoring system (SOM) was collected from the validation cohort, while the individual score and total score for every participant were calculated automatically in an Excel sheet. The performance of the total score was assessed using ROC curve analysis. The AUC above 0.6 was considered acceptable. The logistic regression analysis was finally performed to calculate the equation of death probability from total SOM. All tests were two-sided and a p-value of less than 0.05 was considered significant.

### Institutional review board statement

The study was conducted in accordance with the Declaration of Helsinki, and approved by after approval by the Research Ethics Committee of Faculty of Pharmacy, Tanta University (REC-TP code: TP/RE/012-21P-005) and the ethical committee office of the Medical Military Academy.

### Informed consent statement

Informed consent was obtained from all subjects involved in the study.

## Results

### Patients' characteristics

The total number of data points was 316, and the mortality rate of patients with COVID-19 pneumonia was 28.5%. As shown in Table 1, the basic criteria of both the survival and mortality groups were presented. One third of cases were male, and more than half of them were older than 60 years old. The most frequent comorbidities were DM and HTN, and nearly all patients were presented with cough and dyspnea. However, fever was a significant cardinal sign that correlated with mortality. The duration of complaints in the mortality group was significantly shorter than the survival one, as more than half of them came with a complaint history of less than 4 days. In the mortality group, the CT-SS was significantly higher and more than 2/3 of cases had severe and very severe degrees of CT scores (> 20). Moreover, neutrophil (%), NLR and PLR, as well as AST, urea, creatinine and IL-6 were significantly higher in the mortality group.

**Table 1:** Socio-demographic data and clinical characteristics.

Factors^a^	Died (n = 90/316)		Survived (n = 226/316)		p-value
Sex (male) (n, %)	69	76.67	161	71.24	**0.32**^**ƪ**^
Age (year) (mean, SD)	65.7	14.9	59.2	15.6	**0.001**^**†**^
**Age group (n, %)**					
≤ 60	21	23.33	102	45.13	** < 0.001 **^**ƪ**^
> 60	69	76.67	124	54.87	
**Co-morbidities (n, %)**					
HTN (yes)	54	60	110	48.67	**0.06**^**ƪ**^
DM (yes)	56	62.22	111	49.12	**0.03**^**ƪ**^
IHD (yes)	8	8.89	24	10.62	0.64^**ƪ**^
HD (yes)	4	4.44	6	2.65	0.41^**ƪ**^
Stroke (yes)	2	2.22	6	2.65	0.82^**ƪ**^
**Presenting symptoms (n, %)**					
Fever (yes)	38	42.22	62	27.43	**0.01**^**ƪ**^
Dyspnea (yes)	90	100	224	99.12	1^**ƪ**^
Cough (yes)	90	100	223	98.67	1^**ƪ**^
Fatigue and headache (yes)	0	0	25	11.06	**0.001**^**ƪ**^
Loss of smell (yes)	46	51.11	98	43.36	**0.21**^**ƪ**^
Loss of taste (yes)	53	58.89	99	43.81	**0.01**^**ƪ**^
GIT symptoms (yes)	54	60	148	65.49	**0.35**^**ƪ**^
Time till hospital admission (days) (mean, SD)	3.39	1.12	4.03	2.08	**0.001**^**†**^
**Time till hospital admission group (n, %)**					
< 4 days	52	57.78	92	40.71	**0.006**^**ƪ**^
≥ 4 days	38	42.22	134	59.29	
Oxygen saturation level (< 92%) (n, %)	90	100	123	54.42	** < 0.001**^**ƪ**^
CT-SS (mean, SD)	27.83	7.05	18.55	6.98	** < 0.001**^**†**^
High CT-SS (> 20) (n, %)	68	75.56	80	35.4	** < 0.001 **^**ƪ**^
**Initial routine lab**					
HB (mean, SD)	13.1	2.1	12.74	2.06	**0.19**^**†**^
TLC (mean, SD)	9.2	6.34	8.27	5.6	**0.24**^**†**^
PLT (mean, SD)	195.5	90.8	208.3	92.7	**0.28**^**†**^
Lymphocytes (%) (mean, SD)	25.7	13.6	29	13.1	**0.28**^**†**^
Neutrophilis (%) (mean, SD)	64.5	26.2	55.3	24.9	**0.008**^**†**^
NLR (mean, SD)	6.22	3.37	3.31	1.46	**0.001**^**†**^
High-NLR (> 3) (n, %)	51	56.67	93	41.15	**0.01 **^**ƪ**^
PLR (mean, SD)	18.3	3.3	12	2.5	**0.02**^**†**^
ALT (mean, SD)	31.3	17.7	37.7	13.8	**0.14**^**†**^
AST (mean, SD)	43.3	27.9	34.8	23	**0.02**^**†**^
Urea (mean, SD)	64.9	25.1	51.4	22	**0.008**^**†**^
Creatinine (mean, SD)	1.30	0.74	1.27	0.65	0.72^**†**^
IL-6 (mean, SD)	281	85	84	19	**0.02**^**†**^
**Treatment (n, %)**					
Iverzine (yes)	15	16.67	15	6.64	**0.009**^**ƪ**^
Chloroquine (yes)	12	13.33	35	15.49	0.62^**ƪ**^
Remdesivir (yes)	25	27.78	99	43.81	**0.008**^**ƪ**^
SL (yes)	70	77.78	190	84.07	**0.18**^**ƪ**^
Tocilizumab (yes)	45	50	77	34.07	**0.009**^**ƪ**^

DM: Diabetes mellitus, HTN: Hypertension, IHD: ischemic heart disease, HD: hepatic disease, DOC: duration of complain, CT-SS: Ct severity score, TLC: total leucocytes count, PLT: platelets, NLR: neutrophil lymphocytic ration, PLT, platelets lymphocyte ratio, ALT: Alanine transaminase, AST: Aspartate transaminase, IL-6: Interleukin-6, SL: Sofosbuvir/Ledipasvir, †: Independent t-test, ƪ: Chi-square test.

Significant values are in bold.

^a^The parametric data presented as mean and stander deviation (SD), and categorical data as number and percentage (%).

### Predictors of mortality

After selection of all factors in univariate analysis with a p-value ≤ 0.35, multivariate analysis with different approaches was applied to select the most significant factors that affected in-hospital mortality. As shown in Table 2, being elderly (> 60 years), having a shorter duration of complaint, having a high NLR, and having a higher CT-SS (> 20) were all significant independent predictors of mortality (P < 0.05 for all).

**Table 2:** Predictors of mortality using multivariate logistic regression model with different technique.

Factors	Coeff	OR	95% CI	*p-*value
**A-Adjusted model**				
Time till hospital admission (days)	− 0.41	0.67	(0.4311,1.0314)	**0.04**
HB	0.10	1.10	(0.8476,1.4343)	0.46
TLC	0.02	1.02	(0.9162,1.1357)	0.72
PLR	− 0.02	0.98	(0.9230,1.0316)	0.38
ALT	− 0.03	0.97	(0.9459,0.9980)	**0.01**
AST	0.03	1.03	(1.0004,1.0541)	**0.05**
Urea	0.00	1.00	(0.9955,1.0141)	0.33
IL-6	0.00	1.00	(0.9994,1.0016)	0.35
NLR	0.26	1.30	(1.0717,1.5795)	**0.01**
Old-age (> 60)	1.41	4.11	(1.4187,11.8858)	**0.01**
Sex (male)	− 0.05	0.95	(0.3206,2.7993)	0.92
HTN (yes)	− 0.59	0.56	(0.1987,1.5525)	0.26
DM (yes)	0.61	1.84	(0.6917,4.8885)	0.22
Fever (yes)	0.53	1.70	(0.6471,4.4487)	0.28
Loss-of-smell (yes)	0.34	1.40	(0.4519,4.3542)	0.56
Loss-of-taste (yes)	− 0.26	0.77	(0.2427,2.4284)	0.65
GIT-symptoms (yes)	− 0.55	0.58	(0.1962,1.7058)	0.32
High-CT-SS (> 20)	1.43	4.17	(1.6055,10.8530)	** < 0.001**
Iverzine (yes)	1.00	2.71	(0.7032,10.4537)	0.15
Remdesivir (yes)	− 1.33	0.27	(0.0972,0.7265)	**0.01**
SL (yes)	− 1.46	0.23	(0.0377,1.4235)	0.12
Tocilizumab (yes)	0.99	2.69	(1.0421,6.9419)	**0.04**
**B-Forward selection**				
Time till hospital admission (days)	− 0.38	0.68	(0.4593,1.0197)	**0.05**
ALT	− 0.03	0.97	(0.9510,0.9992)	**0.01**
AST	0.02	1.02	(0.9969,1.0465)	0.09
NLR	0.21	1.23	(1.1034,1.3703)	** < 0.001**
Old-age (> 60)	1.31	3.70	(1.3912,9.8483)	**0.01**
High-CT-SS (> 20)	1.62	5.04	(2.1200,11.9771)	** < 0.001**
Iverzine (yes)	1.14	3.11	(0.9641,10.0462)	0.06
Remdesivir (yes)	− 1.45	0.23	(0.0923,0.5921)	** < 0.001**
SL (yes)	− 1.24	0.29	(0.0592,1.4261)	0.14
Tocilizumab (yes)	0.95	2.57	(1.1177,5.9218)	**0.02**
**C-Backward elimination**				
Time till hospital admission (days)	− 0.33	0.72	(0.4960,1.0343)	**0.05**
NLR	0.17	1.19	(1.0732,1.3141)	** < 0.001**
Old-age (> 60)	1.47	4.36	(1.6666,11.3903)	** < 0.001**
High-CT-SS (> 20)	1.70	5.49	(2.3476,12.8491)	** < 0.001**
Iverzine (yes)	1.10	3.01	(0.9728,9.2905)	0.06
Remdesivir (yes)	− 1.30	0.27	(0.1137,0.6486)	** < 0.001**
Tocilizumab (yes)	0.92	2.51	(1.1144,5.6454)	**0.02**

Goodness of fit test: Hosmer–Lemeshow, P > 0.05 for all models, coeff: coefficient, OR: Odd ratio, CI: confidence interval, P < 0.05 considered significant, the sign before coefficient number denote the direction of relationship.

Significant values are in bold.

### Score model formation and its performance

**Figure 2:**
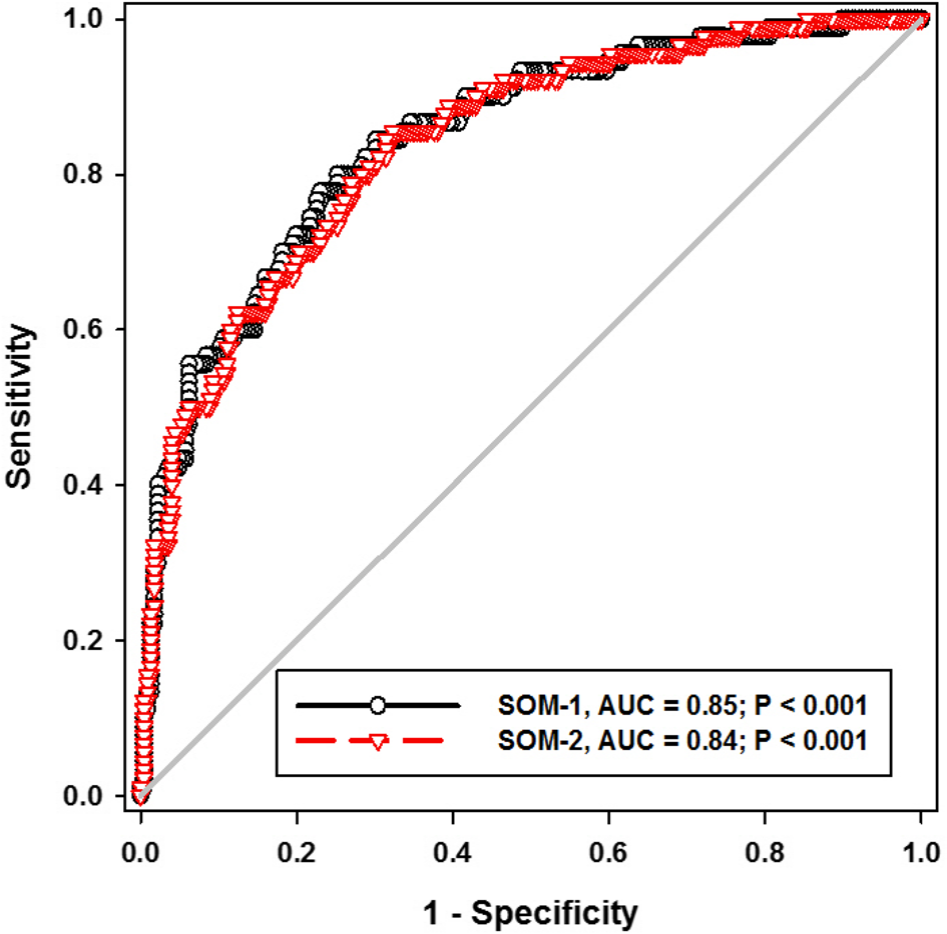
ROC curve for the proposed score of mortality (SOM) (SOM-1: score of mortality No-1, SOM-2: score of mortality No-2, AUC: area under curve, P considered significant if < 0.05).

After collecting the predictors from the previous step, a multiple logistic regression analysis were applied to weight the factors and forming the scoring number based on both WS and Coefficient number. As shown in Table 3, two different scores could be used; the first one (SOM-1) including tocilizumab, and the second one (SOM-2) without it. However, both of them showed insignificant differences in discriminating in-hospital mortality (Fig. 2). Because the AUC was 85% and 84%, respectively, with P = 0.001 for both, SOM-1 was chosen for further validation.

**Table 3:** Development of scoring system based on best predictors for mortality.

Factors	Coeff.	OR	WS	95% CI	*p-*value	Score
**Model for score: SOM-1**						
Time till hospital admission (days)	− 0.60	0.55	15.88	(0.4081,0.7368)	< 0.001	− 3
NLR	0.17	1.18	18.71	(1.0952,1.2733)	0.001	1
Old-age (> 60)	1.28	3.58	13.45	(1.8107,7.0752)	< 0.001	5
High-CT-SS (> 20)	1.66	5.26	27.33	(2.8244,9.8121)	< 0.001	14
Remdesivir (yes)	− 1.17	0.31	11.35	(0.1582,0.6140)	< 0.001	− 4
Tocilizumab (yes)	0.52	1.68	2.81	(0.9164,3.0898)	0.09	1
Constant	− 1.06	0.34	3.12			14
**Model for score: SOM-2**						
Time till hospital admission (days)	− 0.59	0.54	15.92	(0.4093,0.7372)	< 0.001	− 4
NLR	0.17	1.18	19.64	(1.0996,1.2782)	< 0.001	1
Old-age (> 60)	1.3	3.87	15.42	(1.9710,7.6171)	< 0.001	8
High-CT-SS (> 20)	1.70	5.48	28.85	(2.9467,10.1984)	< 0.001	18
Remdesivir (yes)	− 1.11	0.32	10.62	(0.1669,0.6402)	0.001	− 5
Constant	− 0.98	0.37	2.70			18

Goodness of fit test: Hosmer–Lemeshow, P > 0.05 for all models, coeff: coefficient, OR: Odd ratio, CI: confidence interval, P < 0.05 considered significant, the sign before coefficient number denote the direction of relationship, WS: Wald statistics.

### Assessment of the new score: validation and prognostic utility

About 327 patients were subjected to score validation; the descriptive statistics of the validation cohort were summarized in Table 4, in which the mortality rate was 28.74%.

**Table 4:** Characters of validity cohort.

Factors	Died (n = 94/327)		Survived (n = 233/327)	
Age (> 60)	75	79.79	129	55.36
DOC (< 4)	41	43.62	107	45.92
CT-SS (> 20)	54	57.45	58	24.89
NLR (> 3)	63	67.02	98	42.06
Remdisivir (yes)	17	18.09	67	28.76
Actemera (yes)	49	52.13	55	23.61

The parametric data presented as mean and stander deviation (SD), and categorical data as number and percentage (%).

**Figure 3:**
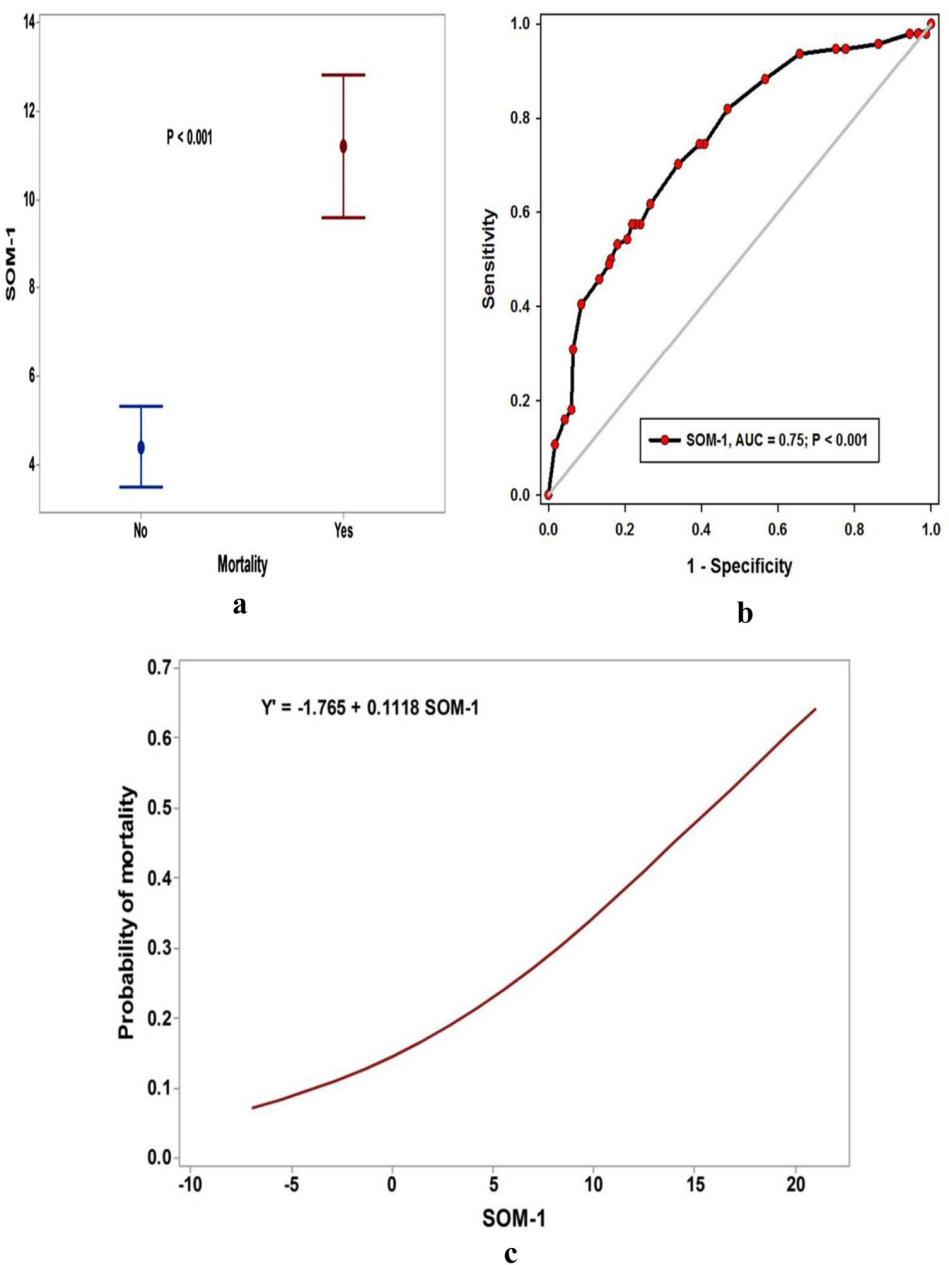
Score of mortality (SOM) in validating cohort.

Moreover, Fig. 3a showed the median (IQR) value of SOM-1 was significantly higher in the mortality group; (13.5 (3–18) than the survival one; 2 (− 0.5–7)), P < 0.001. The prognostic utility of SOM-1 was so good; the AUC was 75%, P < 0.001 (Fig. 3b), and at cutoff values of above 5 and 16.5, the sensitivity and specificity were above 90%, respectively (Table 5). The probability of mortality increased with every unit increase in the SOM-1 (Fig. 3c).

**Table 5:** Performance of SOM-1 in predicting in-hospital mortality.

Cutoff >	Sensitivity (%)	95% CI	Specificity (%)	95% CI	PV+ (%)	PV− (%)
5	94	0.8662 to 0.9762	34	0.2826 to 0.4082	24	96
16.5	40	0.3042 to 0.5105	91	0.8705 to 0.9468	51	87

PV + : Positive predictive value, PV−: negative predictive value.

## Discussion

The current study used the observational data from patients with COVID-19 pneumonia to develop a simple predictive model for further building a scoring system that easily calculates the probability of in-hospital mortality. The algorithms of the predictive models were applied to 316 patients and included simply collected historical, clinical, and laboratory data on the day of admission, which gave our model of prediction the capability to be applied in other medical sectors with an affordable set of data collection and less sophisticated investigation. However, so many scoring systems have been developed since the start of the pandemics to predict different outcomes related to COVID-19 disease. Their sensitivity and specificity ranged between 70 and 100%^37–39^, and the most effective study recorded a model with excellent validity (AUC = 93.8%)^20^.

The present score, besides its easy and manual calculation from simple data, had the capability for prognosis prediction; the utility of the score was very good enough to be accepted, hence the AUC of 85% in the training cohort and still good when applied to the validating cohort (AUC of 75%), which indicates how much the stability of the score in predicting the disease prognosis made the treatment strategy more powerful if it was applied as early as possible for a much better outcome. Moreover, during building the model, we entered medication that was used in local treatment protocol in response to laboratory results to estimate if these factors were implicated in the final outcome or not. We reported that remdesivir and tocilizumab were significantly correlated with in-hospital mortality. Therefore, two scoring systems were calculated with tocilizumab (SOM-1) and without tocilizumab (SOM-2), with an insignificant difference between both of them. The accuracy was 85 and 84%, respectively (Fig. 2). For that reason, SOM-1 was the choice for further external validity. Another concern that made our newly formed score more stable was that we depended on both the coefficient number and Wald statistic number from the logistic regression model to weight the predictors.

The attendance results did not find any surprising factors that affected the mortality except the duration before hospital admission. Hence, we found that patients with a shorter duration of less than 4 days before admission died, which made the explanation more difficult, and it could be related to the state of denial that the patients caught the disease and suffered from silent prolonged hypoxia. Surprisingly, only one study includes this historical item in the predictive model of COVID-19-related mortality. Henderson et al*.* reported that a shorter time from symptom onset to hospitalization is associated with a more serious disease and higher mortality^40^. On the other hand, old age was correlated with bad prognosis, which came in consistence with other reports^41–46^.

Despite the fact that many studies have pointed to the importance of male sex and associated comorbidity in poor prognosis^45–49^, our study did not find that link after multiple filtration of logistic regression modeling, and a recent study supported that^50^. Furthermore, the severity level of lesion in HRCT was one of the most reliable predictors; thus, it was associated with a poor prognosis, despite the fact that a few studies used that factor to predict in-hospital mortality outcome^50–52^.

Our study introduced some treatment medications like iverzine^53,54^, sofosbuvir/ledipasvir^55^, remdesivir, and tocilizumab to be estimated as predictive factors for mortality in a regression model. iverzine was used following our national COVID-19 management guidelines in its old versions^53,54^, however it is deleted from the recent version^56^. Only remdesivir and tocilizumab continued to be linked with mortality after multiple filtrations of the predictors. Hence, remdesivir was found to be protective against a bad prognosis, and early administration could inhibit the replication of viruses and decrease the viral load; it could also counter the process of pathology inside the lung parenchyma that finally led to improvement in the lung lesion^57^.

On the other hand, our data showed that tocilizumab increased the likelihood of mortality two times more, which could be due to limiting the use of that medication in patients with severe disease, so the risk of its link to a bad prognosis became much higher. Additionally, tocilizumab had been prescribed in COVID-19 patients with an established higher level of IL-6, denoting that a cascade of cytokines storm had been started^58–60^. Although a pooled analysis of systematic reviews on tocilizumab and mortality outcomes found that it was not only protective against bad outcomes, it was also significantly linked to post-drug infection, which led to a poor prognosis from super infection^61^. That fact could explain the present finding and draw attention to the limited power of using biological therapy in treatment. Nevertheless, several newly published randomized controlled trials (RCTs)^62–70^ and meta-analyses^71–73^ of RCTs have investigated the effects of TCZ as an adjunctive therapy in patients with COVID-19 but have reported inconsistent results. Moreover, there are increasing number of newly available studies regarding TCZ treatment for COVID-19. Therefore, there are still limited real-world data about the effect of TCZ on inflammatory activity in COVID-19 patients^74^.

## Limitations

Even though our study was the first of its kind that was proposed in Egypt, which is a developing country with limited resources, it highlighted the ability of simple data to predict the outcome of COVID-19 patients. However, the study showed some limitations; the first was the types of study design; hence, the main issue with retrospective cohort type studies was the effect of confounders, which could influence the final outcome in an unpredicted way. The second limitation was the single-centre study, which increased the demand for further external validation from other centers. Additionally, the third point was that the study excluded younger individuals < 18 years old during recruitment, which may be an area of future interest. Moreover, the score was constructed for only COVID-19 patients, which made the comparison with other alternative scores much more difficult to apply.

## Conclusion

The constructed score (EGY.Score) from the observational data could predict the prognosis of patients with COVID-19 pneumonia, which may possibly be used to adjust the management intervention for further gain of a desirable outcome.

## Supplementary Information

Supplementary Figure 1.

## Data Availability

All data generated or analyzed during this study are included in this published article and supplementary material.
