# Supplementary material for: Development and validation of a predictive scoring system for in-hospital mortality in COVID-19 Egyptian patients: a retrospective study
Source: Sci Rep. 2022 Dec 26;12:22352. doi: 10.1038/s41598-022-26471-w (PMC9791155; doi:10.1038/s41598-022-26471-w)
Supplement: Supplementary file 1 — Supplementary Figure 1. [file 41598_2022_26471_MOESM1_ESM.docx]

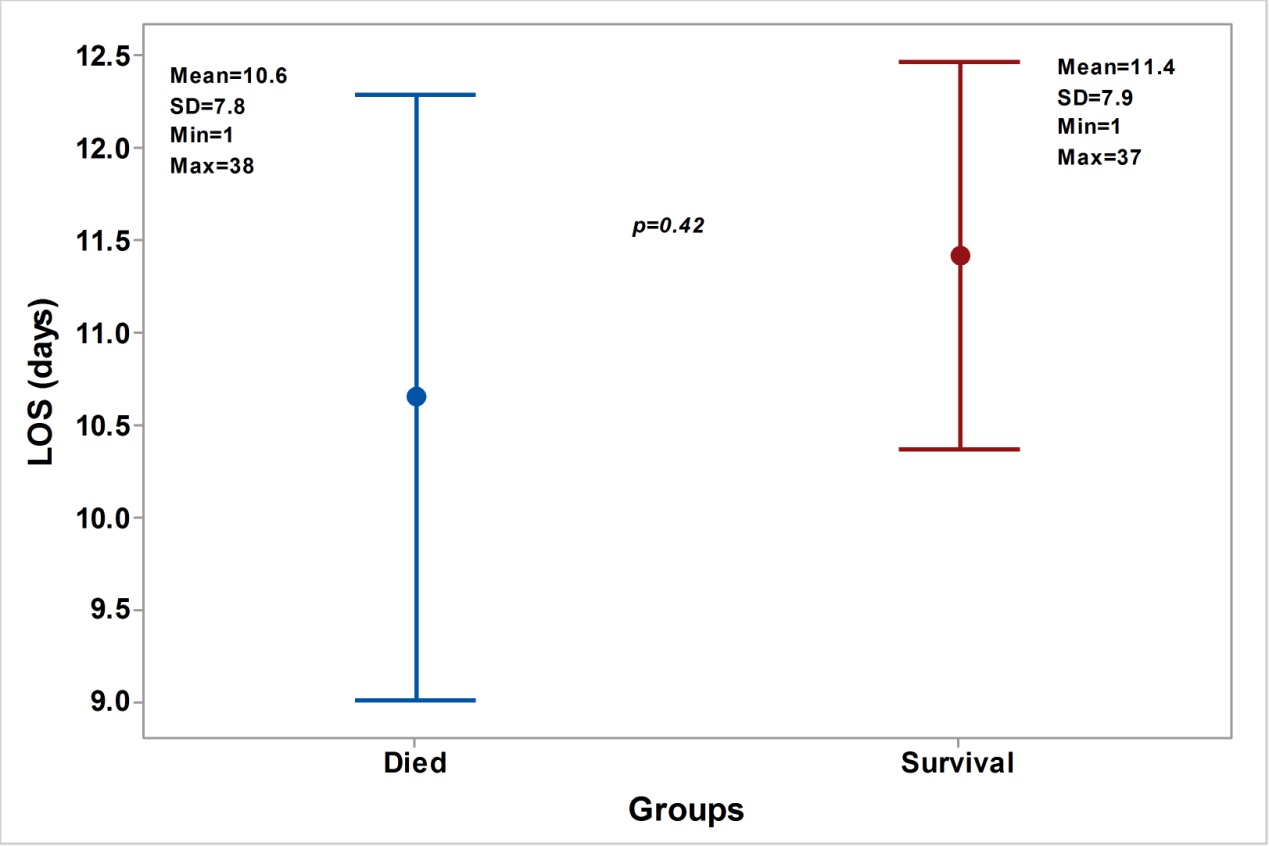


**Supplementary Fig.1:** Length of stay (LOS) in hospital for patients with COVID-19 pneumonia. The test of significant: independent t-test, *P* < 0.05 considered significant.
